# Promoting the use of evidence in health policymaking in the ECOWAS region: the development and contextualization of an evidence-based policymaking guidance

**DOI:** 10.1186/s12992-020-00605-z

**Published:** 2020-08-06

**Authors:** Chigozie Jesse Uneke, Issiaka Sombie, Ermel Johnson, Bilikis Iyabo Uneke, Stanley Okolo

**Affiliations:** 1 African Institute for Health Policy and Health Systems, Ebonyi State University, CAS Campus, Abakaliki, PMB 053 Nigeria; 2 West African Health Organisation, 175, Avenue Ouezzin Coulibaly, Bobo Dioulasso, 01 01 BP 153 Burkina Faso

**Keywords:** Evidence, Health, Policymaking, West Africa, Guidance

## Abstract

**Background:**

The Economic Commission of the West African States (ECOWAS), through her specialised health Institution, the West African Health Organization (WAHO) is supporting Members States to improve health outcomes in West Africa. There is a global recognition that evidence-based health policies are vital towards achieving continued improvement in health outcomes. The need to have a tool that will provide systematic guide on the use of evidence in policymaking necessitated the production of the evidence-based policy-making (EBPM) Guidance.

**Methods:**

Google search was performed to identify existing guidance on EBPM. Lessons were drawn from the review of identified guidance documents. Consultation, interaction and interviews were held with policymakers from the 15 West African countries during WAHO organized regional meetings in Senegal, Nigeria, and Burkina Faso. The purpose was to elicit their views on the strategies to promote the use of evidence in policymaking to be included in the EBPM Guidance. A regional Guidance Validation Meeting for West African policymakers was thereafter convened by WAHO to review findings from review of existing guidance documents and validate the EBPM Guidance.

**Results:**

Out of the 250 publications screened, six publications fulfilled the study inclusion criteria and were reviewed. Among the important issues highlighted include: what evidence informed decision-making is; different types of research methods, designs and approaches, and how to judge the quality of research. The identified main target end users of the EBPM Guidance are policy/decision makers in the West African sub-region, at local, sub-national, national and regional levels. Among the key recommendations included in the EBPM Guidance include: properly defining/refining policy problem; reviewing contextual issues; initiating policy priority setting; considering political acceptability of policy; commissioning research; use of rapid response services, use of policy advisory/technical/steering committees; and use of policy briefs and policy dialogue.

**Conclusion:**

The EBPM Guidance is one of the emerging tools that can enhance the understanding of evidence to policy process. The strategies to facilitate the use of evidence in policymaking outlined in the Guidance, can be adapted to local context, and incorporated validated approaches that can be used to promote evidence-to-policy-to-practice process in West Africa.

## Introduction

The Economic Commission of the West African States (ECOWAS) has over the years encouraged member states to increase investment in the health sector and through her specialised health institution, the West African Health Organization (WAHO) has supported the efforts of members States to improve health outcomes in the region [1]. The poor maternal and child health outcomes in West Africa have been shown to be associated with diverse contextual and health systems factors inherent within the sub-region. These factors include, culture, knowledge of risks and the status of women, geographic distance of health centres, services delivery organisation, the availability and ability of health services, and the quality of care, and these all act together to exacerbate the poor health outcomes in the region [1, 2]. According to Agyepong et al. [2], the West African countries are in dire need of increased investment in health interventions and health systems strengthening approaches that are evidence-based.

Currently there is a global recognition that strong and effective health systems and health policies that are evidence-based in their operations are vital to achieve continued improvement in health outcomes in an efficient and equitable manner [3, 4]. According to World Health Organization (WHO), better use of evidence in development policy making can save lives through more effective policies that respond to scientific and technological advances, use resources more efficiently and better meet citizens’ needs [5].

In addition to scientific evidence which typically includes research/surveys, quantitative/statistical data, qualitative data, and analysis, evidence can also include economic, attitudinal, behavioural and anecdotal information, along with knowledge and opinions of experts, as well as judgements, insight/experience, history, analogies, local knowledge and culture [6]. In a previous report, Bowen and Zwi [7], noted that evidence encompasses research, and may include opinion and views of individuals or groups, and results of consultative processes. It is therefore imperative to elicit the views of policy actors and information gathered through consultative engagements involving critical stakeholders as part of evidence-to-policy process. Evidence based on scientific research is combined with other forms of information to provide evidence for policy development and practice [6]. This approach can help to contextualize the policymaking process and most likely work well in West Africa. It has been reported that combining different forms of evidence creates and acknowledges the context within which knowledge exists and within which it is understood [8]. Adequate combination of both scientific and other forms of evidence is therefore the hallmark of evidence-informed decision making, a process that must be employed if effective and result oriented health policies are to be achieved in West Africa.

Poorly-informed decision-making is partly responsible for the weakness of the health systems of most West African countries [2]. In a previous report Oxman et al. [9], noted that a poorly-informed decision-making particularly in LMICs is one of the reasons why services sometimes fail to reach those most in need, why health indicators become off-track and why many countries fail to meet health targets. Nevertheless, it is important to re-emphasize that the process of incorporating evidence into policy making i.e. bridging the know-do-gap, is neither a simple nor straightforward venture. According to Jones and Walsh [10], the integration of evidence into policy decision making is a complex process of multiple, frequently competing and/or intertwined sets of influences in which evidence plays just one of many roles. Strydom et al. [6] reiterated that the policymaking context is full of political, ideological and economic factors that influence policy development and decision-making, often at the expense of scientific evidence, and that decision-makers and policymakers in many cases source information with a particular agenda in mind.

A number of recent studies have stressed the urgent need for increased understanding of the complexities of the evidence to policy process among policymakers in West Africa and also a need to have a guidance on evidence-based policymaking [1, 2, 11]. Guidance documents with specific focus on evidence-based policymaking or evidence-informed policymaking are essentially scarce. However, in recent times, the need for the production of guidance on evidence-based policymaking is increasingly being recognized worldwide. The process of policymaking remains very complex, necessitating a step by step approach towards achieving evidence-based policymaking. As a part of its mandate to promote evidence-to-policy-to practice process in West Africa, the WAHO commissioned a project to develop an adapted guide for the use of evidence in developing and implementation of health policies, plans and protocols in the ECOWAS region. The objective of this report is to describe the process of development and content of the evidence-based policy-making (EBPM) guidance designed for the promotion of the use of evidence in health policymaking in West Africa.

## Methods

### Setting

The West African sub-region is made up of 15 countries (Benin, Burkina Faso, Cape Verde, Cote d’Ivoire, The Gambia, Ghana, Guinea, Guinea Bissau, Liberia, Mali, Nigeria, Niger, Sierra Leone, Senegal and Togo). It is geographically bounded by the Atlantic Ocean in the west and by the Gulf of Guinea in the south and is characterised by a very rich ethnic, religious and social diversity [12]. According to the United Nations Development Programme report, most of the West African countries are classified as poor and their economies are not very well developed or diversified with the Human Development Index (HDI) rank among the poorest in the world [13].

The West African sub-region with a population of more than 357million (about 1/3 of entire African population) is one of the regions in Africa with under-performing health systems and unacceptable health outcomes [13, 14]. Many of the West African countries are among the low-and-middle income countries (LMICs) world-wide with the lowest life expectancy [13]. The maternal mortality ratio (MMR) of some of the countries in the region are among the highest in the world. These include, Sierra Leone (1360/100,000), Nigeria (814/100,000), Liberia (725/100,000) and The Gambia (706/100,000) [14]. Some West African countries record under-five mortality rates (U5MR) which are among the highest in the world, and these include Sierra Leone (118/1000), Mali (114/1000), Nigeria (108/1000) and Benin (100/1000) [14].

Defor et al. [15] noted that the complexity of the sub-region are layered in traditional, ethnic, religious and language diversity, which is further heightened by the colonial legacy of fragmentation of the sub-region by official language into Anglophone, Francophone and Lusophone. Because the complexities in the social structure of West Africa to a large extent influence the policymaking process in the sub-region, a policymaking guide that will take contextual issues into consideration becomes imperative.

### Review of previous guidance publication focusing on evidence use in policymaking

A Google search was performed in May 2019 to identify existing guidance on evidence-based policymaking within the previous 20 years. The search was executed using appropriate combination of key words such as: *guidance, guidelines, evidence-based, evidence-informed, policymaking*, *decision-making, health policy*. A total of 250 publications were screened using the following key inclusion criteria: (i). must be focused on health policymaking, (ii). must be a guidance/guideline document, (iii). must be intended for use by policymakers/decision makers, (iv). must outline step by step procedure for evidence-based policymaking/decision making. Following the screening, the publications that fulfilled the inclusion criteria were selected for the study.

### Consultation with west African policymakers

Authors consulted and interacted with some senior health policymakers from various West African countries. These policymakers from the 15 West African countries were engaged into discussions and interviews during WAHO organized regional meetings in Senegal, Nigeria, and Burkina Faso prior to a Validation Meeting held in Senegal in July 2019. This method has been used successfully in previous studies to obtain information from policymakers [16, 17]. Others were consulted via telephone calls and email. The discussions and interview centred on the following: (i). The necessity of the EBPM Guidance; (ii). Intended users of the EBPM Guidance; and (iii). Recommended strategies to promote the use of evidence in policymaking to be included in the EBPM Guidance. Responses from the policymakers were noted and formed a part of the information collected and used for the development of the Guidance.

### Validation of the guidance by west African policymakers

Following the production of a draft EBPM Guidance, both internal and external reviews were conducted by WAHO. A revised version was produced, and thereafter WAHO convened a Guidance Validation Meeting in July 2019 in Senegal. The objectives of the meeting were:

(i). To raise awareness on the implementation of the regional resolution on the use of evidence.

(ii). To share the findings of the review of previous guidance publication performed in May 2019 and findings of study on the use of evidence in decision making in West Africa.

(iii). To validate the Regional Guide on the use of evidence in the ECOWAS region.

The meeting brought together heads of maternal and child health programmes, as well as research managers from the Ministries of Health of ECOWAS member countries and key partners. Prior to the meeting the revised EBPM Guidance was circulated to all invited participant to study and make inputs to be discussed as the validation meeting. During the meeting the revised EBPM Guidance was presented and subjected to group work. More suggestions and recommendations for the improvement of the Guidance were received and discussed to arrive at a consensus on the final version of the Guidance.

## Result

### Description of previous guidance publications focusing on evidence use in policymaking

Out of the 250 publications screened, six publications fulfilled the study inclusion criteria and were reviewed. Table 1 summarizes the characteristics of the reviewed guidance documents on evidence-based policymaking [18–23]. Among the important issues highlighted include: what evidence informed decision-making is; different types of research methods, designs and approaches, and how to judge the quality of research; key stages of the public policy-making process and evidence use; facilitators and barriers to evidence use in policy-making; linking evidence needs to policy priorities; context of public policy-making; key stages of the policy-making process and evidence use, and legislators use of research evidence in public health policymaking. Lessons were drawn and insights gained from these six guidance/guideline documents which were used in developing the present EBPM Guidance.

**Table 1:** Characteristics of existing guidance documents on evidence-based policymaking

Author [Reference]	Year of publication	Title	Target users	Main focus of Guidance
Blanchet et al. [18]	2018	Using Research Evidence in the Humanitarian Sector: A practice guide	Policymakers, practitioners	(i). What evidence informed decision-making is, (ii). developing and implementing a new intervention, (iii). creating a theory of change, (iv). different types of research methods, designs and approaches, and how to judge the quality of research.
Ministry of Health, Malawi [19]	2016	Guidelines for Evidence Use in Decision-Making in the Health Sector	Policymakers	(i). Foundation of Public Policy-Making, (ii). theory in public policy-making, (iii). key stages of the public policy-making process and evidence use, (iii). characteristics of good public policy-making, (iv). facilitators and barriers to evidence use in policy-making,
Wills et al. [20]	2016	Guidelines and good practices for evidence-informed policy-making in a government department	Policymakers	(i). Using a broad definition of ‘robust evidence’, (ii). linking evidence needs to policy priorities, (iii). linking an evidence-informed approach with business planning, budgeting and reporting, (iv). ensuring evidence processes are inclusive and participatory
Breckon et al. [21]	2016	Using Research Evidence: A Practice Guide	Decision-makers in government, voluntary organizations	(i). The four elements of evidence-based management (ii). reasons for needing evidence and creating a theory of change, (iii). how to judge the quality of research and where to look for evidence (iv). how to communicate findings
Ministry of Health, Kenya [22].	2015	Guidelines for evidence use in policy-making	Policymakers	(i). Context of public policy-making, (ii). key stages of the policy-making process and evidence use, (iii). facilitators and barriers to evidence use in policy-making, (iv). health policy development process, accessing evidence for policy-making
The Council of State Governments [23]	2008	State Policy Guide: Using Research in Public Health Policymaking	Policymakers, legislators	(i). Legislators use of research evidence in public health policymaking (ii). understanding public health research: key facts and terms (iii). how researchers measure success of policies and programs? (viii). using research results in policymaking, and drafting public health legislation

### Identified intended users of the guidance

The identified target end users of the EBPM Guidance are policy/decision makers in the West African sub-region at local, sub-national, national and regional levels. These include elected policymakers (eg., Presidents, Governors, members of parliament), appointed policymakers (e.g., Ministers, Commissioners, Directors-General), career policymakers (e.g., Heads of departments, Programme managers, senior officials) and civil sector policymakers (e.g., Traditional rulers, Religious leaders, leaders of NGOs/CSOs).

### Recommended strategies to promote the use of evidence in policymaking

Following the Guidance validation meeting, the recommendations received were categorized into fourteen strategies (Table 2). These strategies were systematically arranged to form the protocol of the EBPM Guidance. The strategies are not necessarily chronological, but the step-by-step implementation of the strategies will depend on the country specific context. Participants considered the recommendations as fundamental principles that can potentially promote evidence-to-policy process within the West African sub-region. Participants agreed that the EBPM Guidance be contextualized in each country in line with the peculiarities of the countries.

**Table 2:** Recommended strategies for the EBPM Guidance to promote the use of evidence in policymaking for West Africa

1.	Properly define/refine the policy problem, state policy questions
2.	Identify and review existing similar policies
3.	Review contextual issues (contextualization)
4.	Initiate policy priority setting
5.	Consider political acceptability of the policy
6.	Access, retrieve, assess, and synthesis evidence
7.	Commission research/engage researchers/ co-produce evidence and policy/ use rapid response services
8.	Perform stakeholders’ analysis & Convene stakeholders’ engagement event
9.	Use of policy advisory/technical/steering committees
10	Develop policy briefs and undertake policy dialogue
11.	Draft the policy document
12.	Subject the policy document to internal and external review
13.	Ensure official endorsement of the policy by government
14.	Institute monitoring, evaluation and review mechanism for the policy

## Discussion

According to Bosch-Capblanch et al. [24] and other authors [25, 26], an evidence-informed health systems and policy guidance tackles health sector problems through the following four important strategies: (i). framing health systems problems; (ii). systematically retrieving, translating, and packaging the best available evidence, (iii). using this evidence to recommend and formulate—in a deliberative process—options to inform policy-making; (iv). providing insights on the strategies that can be followed in order to implement and evaluate policy. The present EBPM Guidance was tailored in line with these four strategies and also followed a systematic approach in its design. The EBPM Guidance is adapted to West African contexts and was validated using standard scientific methods, to consider all the available evidence and to assess its quality. Local factors that may influence the effects of all options recommended to address their feasibility were taken into account in its development. The production of the EBPM Guidance is largely based on knowledge translation approaches that bridge the gap between research evidence and its application to policy-making [27, 28]. Consequently, the EBPM Guidance is considered a very useful tool to decision making process, as it incorporates the complex interrelations of the West African health systems components, and the numerous contextual factors that may influence the effectiveness of policies [24].

### Intended users of the EBPM guidance

The main target end users of the EBPM Guidance are policy/decision makers in the West African sub-region at local, sub-national, national and regional levels. These were classified into elected policymakers, appointed policymakers, career policymakers and civil sector policymakers. This is consistent with a previous report by Bammer et al. [29]. The Guidance is specifically tailored to meet the policymaking needs of the West African policymakers. In addition to these intended users, the Guidance can be of practical benefit to other persons or groups interested in improving their knowledge and skill in evidence informed policymaking.

### Recommended strategies for the EBPM guidance

#### Properly define/refine the policy problem, state policy questions

The foundation for evidence-informed policymaking is proper definition of the policy problem or policy question. It is important to have a clear and unambiguous perspective of the policy objectives before moving to the next stages of the policy development. It will certainly be very difficult to identify the correct and relevant evidence if the policy questions are not well defined. According to the Malawi’s Ministry of Health Guidelines for Evidence Use in Policy-making [19], being clear on the policy issue calls for a good understanding of where the issue lies in the policy-making process/cycle, i.e.: agenda-setting stage, policy formulation stage, policy implementation stage, and policy evaluation stage.

Proper definition and refining of policy problems/questions may require extensive interaction with key stakeholders and citizens affected (or to be affected) by the policy issue. It may also require review of literature and careful/objective assessment of prevailing situation and circumstances to determine if the issue is of serious public health concerns and merits the investment of resources (time, money, man power etc) to move to other stages of policy development.

#### Identify and review existing similar policies

Health policies may be developed for any one of the following reasons: when there is a problem or situation that requires to be addressed and no existing policy to handle that; when there is outdated/obsolete, harmful, unimplementable or ineffective policy that requires reviewing; and for the purpose of strengthening the implementation of a good policy to continue to generate the expected outcomes. Whatever the situation, as part of the process of evidence-informed policymaking, it is important to identify and review existing similar policies when embarking on policy development. The review may include similar health policies that have been developed in other settings. Reviewing what has been done previously is a vital step towards extracting valuable lessons for the development of a new policy.

In conducting a review of a policy document, the following should be considered:

(i). Relevance of the health policy to the health situation prevailing in the population at any point in time.
(ii). Efficiency and effectiveness of the policy in terms of its implementation and performance assessment.
(iii). Impact of the policy on the health outcomes and in terms of achievement of the policy goals and targets.

#### Review contextual issues (contextualization)

It is a well-established fact that policymaking is not a linear process and does not occur in a vacuum. Every society has its own peculiar contextual characteristics which must be understood and taken into consideration during policymaking. It is important to state that no effective policy can be made if contextual factors are not taken into consideration in the policy formulation process. Contextual factors may be environmental or organizational.

In a recent report Peacock and Bentley [30], noted that contextual factors such as different sources and types of evidence used, organizational context and decision-making structures, and the wider interests of patients, the public and politicians can influence decision making process. They further argued that to better understand these influences, especially in terms of policymaking, one needs to takes a wider view using lenses from a range of disciplines [30]. In another recent report, Mijumbi-Deve and Sewankambo [31] highlighted some important contextual factors that must be taken into account in policy development process including relationship between the producers or suppliers and the end users, network effects, culture referring to the norms and values of the environment, power (perceived and/or actual), and high compatibility with current norms and work processes.

Organizational contextual factors that have been found to influence the policymaking process include internal organizational structure – centralization, complexity, formalization, interconnectedness, organizational slack, and external characteristics of the organization [32]. According to the Guidelines for Evidence Use in Policy-Making published by the Kenyan Ministry of Health [22], for policymaking to be effective, civil servants involved in policy development not only need all the ‘traditional’ attributes (knowledge of relevant law and practice, understanding of key stakeholders’ views, ability to design implementation systems), but they must also understand the context within which they (and the policy) have to work.

In a study in Ghana on the health policy agenda setting and formulation on free antenatal care in government facilities, Koduah et al. [33], observed that contextual factors served as bases for policymaking process and included: political ideology, economic crisis, data about health outcomes, historical events, social unrest, change in government, election year, austerity measures, and international agendas. Authors concluded that influencers of policy agenda setting must recognize that the process is complex and intertwined with a mix of political, evidence-based, finance-based, path-dependent, and donor-driven processes [33]. The contextual factors that are likely to influence a health policy in a given population must be carefully identified and taken into consideration when developing a policy document.

#### Initiate policy priority setting

It is important to initiate a policy priority setting process as a way to further establish the necessity of the development of a particular policy. Priority-setting process has been defined as a programme to generate consensus about a core set of health issues that urgently require attention in order to facilitate policy development [34]. Priority setting strategies in the health sector must account for the fact that resources are limited and that trade-offs are required to decide which health conditions deserve the most attention and which interventions should be used to address them [35, 36]. Therefore, no health policy development should be commenced unless the policy to be developed is of absolute necessity. It must be noted that the policy process is not devoid of competing interests and other potential causes of conflicts. A balanced process for setting priorities can harmonize the competing interests, ground value systems, encourage problem-based learning, resolve conflict, find consensus and ultimately create a set of agreed-upon priorities [34].

Priority setting is one of the biggest challenges faced by policymakers in LMICs including West Africa. In low income countries priority setting is known to be complicated by the burden of underdevelopment as well as by political instability, inadequately developed social sectors, weak institutions, and marked social inequalities [37, 38]. In order to conduct an effective policy priority setting, it is important to take into consideration the key contextual factors (such as the social, economic and political factors) of relevance to priority setting, the people or institutions involved in priority setting, the criteria or values used in decision making and how these were identified, the information or evidence used, and a description of the priority setting process [38].

#### Consider political acceptability of the policy

Policymaking can never be separated from politics. According to Oliver [39], science can identify solutions to pressing public health problems, but only politics can turn most of those solutions into reality. In a classical report entitled: Towards a politics of health, Bambra et al. [40] highlighted the political nature of health in three ways:

(i). *Health is political because, like any other resource or commodity under a neo-liberal economic system, some social groups have more of it than others.*
(ii). *Health is political because its social determinants are amenable to political interventions and are thereby dependent on political action (or more usually, inaction).*
(iii). *Health is political because the right to ‘a standard of living adequate for health and wellbeing’ (is, or should be, an aspect of citizenship and a human right).*

There has been an increasing awareness within research and policy communities that getting evidence including research into policy is not only a technical matter of knowledge translation and exchange, but also a political challenge [41, 42].

According to the Alliance for Health Policy and Systems Research, policy making is a complex and essentially political process that is influenced by several factors [43]. Politics has been defined classically as who gets what, when and how and it affects the origins, formulation, and implementation of public policy in the health sector [44]. Politics dictates the factors that affect policymaking process, for example, who is entitled to services, which are the priority areas, who will provide services, who will be subsidized, and how the budget ought to be allocated and spent [44, 45]. Consequently, one of the first critical issues that must be taken into cognizance in policy development is to ensure that the policy planned has political acceptability. Glassman and Buse [44] had earlier argued that in spite of the acknowledged importance of politics in policymaking, there is also broad agreement that politics and political issues are rarely analyzed and frequently ignored at all stages of the policy identification, development, and implementation process in the health sector.

Consequently, we can categorically state that any planned health policy that fails to align with prevailing political situation and ideology, notwithstanding the use of the best available evidence in its development, is dead on arrival. This is because even the best evidence is frequently trumped by politics which can be the main driver of health policy priorities and reforms [44, 46]. Politics played vital role in the success and failures of some of the policymaking and policy implementation processes recorded in some of the West African countries. In Benin, Dossou et al. [47] assessed user fees for caesarean sections and observed that policy development process suffered from inadequate uptake of evidence largely because the policy content and process were not completely in harmony with political goals. In Côte d’Ivoire, Blau et al. [48] noted that a major success factor of the National Immunization Technical Advisory Group (NITAG) in promoting effective immunization policy was the strong political will from the government.

#### Access, retrieve, assess, and synthesize evidence

After defining policy questions, reviewing contextual issues, initiating policy priority setting and considering political acceptability of the policy, the next critical step towards evidence informed policymaking is to access evidence. This requires knowledge of where to source the right and relevant types of evidence. According to Gurung, [49] evidence can come from different types of policy research including case studies, field experiments, secondary analysis, surveys, review of research, qualitative methods, and cost benefit analysis. The major sources of policy research include: specialized policy unit, official statistics, think tanks, academic communities, traditional knowledge, polls, and media [49]. There are other vital sources of evidence such as those available online such as: African Index Medicus, The Cochrane Library, HINARI, POPLINE, PubMed, Research for Life, WHO, Development Experience Clearinghouse (DEC), Social Systems Evidence, Health Systems Evidence, and Campbell Collaborations [19].

After identifying the relevant sources of evidence, the next step is to search and retrieve the needed evidence to be used in the policy process. In assessing the strength of evidence, it is important to know that all evidence are not of equal strength. Assessing the strength of evidence is a challenging task, and requires the application of technical knowledge and individual judgement [50]. Assessment of the overall strength of a body of evidence with reference to a particular policy is directly linked to the quality, size, consistency and context of the body of the evidence [19]. The synthesis of evidence is very important but even more important is making use of synthesized evidence. In this regard, Donnelly et al. [51], outlined four principles to make evidence synthesis more useful for policy as follows: inclusive, rigorous, accessible and transparent.

#### Commission research/engage researchers/ co-produce evidence and policy/ use rapid response services

In the event of scarcity of evidence especially local or context specific evidence, one effective way to generate policy relevant evidence is for policymakers or the Ministry of Health to commission a research. Researchers can be commissioned by the Ministry of Health to conduct research which will provide specific local or context specific evidence required for the formulation of policy. Because evidence to policy link ought to be a continuous never-ending process, the need for policymakers to engage researchers and work in partnership cannot be overemphasized.

One of the major factors responsible for the problem of translating research evidence into policy is the huge gap existing between researchers and policymakers which results in a lack of proper understanding of the concepts and the application of evidence-informed policymaking by decision makers [52]. Uneke et al. [53] noted that there is a dire need for partnership/collaboration between researchers and policymakers in order to (i). acquaint policymakers of evidence regularly produced by researchers and also to carry researchers along in the policymaking process; and (ii). align researchers more specifically to operational problems inherent in the health systems from the policymaking perspective.

A very vital evidence to policy strategy is the engagement of rapid response services (RRSs) when they are available. RRSs are designed to receive policy and decision questions and respond to these with the best available research evidence in summarized and contextualized forms within short periods of time, for example, less than 28 days [54]. According to Mijumbi-Deve and Sewankambo, [31], RRSs improve the timeliness and relevance of research evidence for policy-making and they are also thought to improve contact and interaction between users and producers of research evidence, the lack of which has been cited as a barrier for the use of research evidence during policy and decision-making. In Burkina Faso, the RRSs facilitated the use of research evidence in policymaking and largely reached the consolidation phase of the institutionalization after project leaders, convinced policy-makers of the importance of the service [55].

#### Perform stakeholders’ analysis & convene stakeholders’ engagement event

Policy actors also known as stakeholders have important role to play in shaping evidence to policy link. To enable the success of evidence informed policymaking, stakeholders’ analysis (SHA) is very imperative. According to Ruhe [56], SHA is designed to provide an organization with information to evaluate and understand stakeholders in term of their relevance to a policy or specific activity of the organization. The use of SHA as a systematic technique for gathering insights relating to a proposed policy formulation, reform, or action is gaining increasing recognition worldwide. This is because SHA gathers these insights by identifying, categorising and analysing individuals or groups that are likely to have a ‘stake’ (be affected by, or have an interest) in a proposed policy [57–59]. SHA should identify actors who are decision-makers and as well as those impacted by the policy issue/action being considered; essentially all whose interests, actions and motivations influence the environment [56]. SHA has been developed to better understand stakeholder power and positions around specific new policies or actions, and assess the likely implications for the acceptability of new policies or interventions [57].

After performing the SHA the next step is stakeholders’ engagement. There is evidence to suggest that stakeholder engagement is the key in both implementation and translational sciences which includes tailoring best evidence for policy and practices for specific populations [60]. In Benin, Houngbo et al. [61], assessed governance of healthcare technology management and noted that interactive and analytical tools of action research can be used to integrate knowledge amongst actor groups, and that the policy development process was characterized as bottom-up, with a central focus on the participation of diverse stakeholders groups. In another study from Benin, Lee et al. [62] while assessing vaccine policy, observed that the enabling change required identifying in-country “champions,” and orchestration of a coordinated set of steps that heavily involve key stakeholders. In Cape Verde, Nabyonga-Orem et al. [63] assessed health policy dialogue and observed that ensuring stakeholder participation, improving stakeholder harmonization and alignment, providing a guiding framework and facilitating stakeholder analysis in policy dialogue offered the opportunity to improve stakeholder participation in policy development and promote aid effectiveness.

#### Use of policy advisory/technical/steering committees

The use of policy advisory/technical/steering committees in promoting evidence to policy process is well established. This mechanism is recommended for use to West African policymakers because of the numerous positive outcomes reported across the sub-region. In Nigeria, Uneke et al. [64] assessed the role of a health policy advisory committee (HPAC) in bridging the divide between research and policy. The HPAC can function as a health coordination mechanism designed to standardize and develop a sector wide approach in the development of evidence-informed health policy and the strategies for implementation [64–66].

Other recommended mechanisms similar to the HPAC which are capable of facilitating evidence to policy process are policy technical groups and policy steering committees. In Côte d’Ivoire, Blau et al. [48] described the tremendous success a National Immunization Technical Advisory Group (NITAG) achieved in promoting evidence to policy process regarding immunization and vaccines. Similarly, in Ghana, Howard et al. [67] examined the functionality of NITAG and noted that the NITAGs had an important and valued role within national immunization decision-making. In Burkina Faso, Keita et al. [68] assessed the role of steering committees (SCs) in the promotion of evidence to policy process and observed that SCs provided technical assistance to researchers during the implementation phase and facilitated the transfer and use of the findings.

#### Develop policy briefs and undertake policy dialogue

The use of policy briefs and policy dialogues are major strategies of achieving evidence to policy link. In Nigeria policy briefs and policy dialogues have been used to promote evidence informed policymaking [69, 70]. Lavis et al. [71], noted that policy brief is an effective evidence-packaging mechanism and a new approach to improving the policy-making process by supporting evidence-informed policy-making.

The use of policy briefs and policy dialogues has been reported in many parts of West Africa as effective mechanisms that greatly promoted the evidence to policy link. In Ghana, Araujo de Carvalho et al. [72] reported that policy briefs were developed and were used to define priority problems and health system responses to ageing and health. In Cape Verde, Nabyonga-Orem et al. [63] observed that policy dialogue offered the opportunity to improve stakeholder participation in policy development and promoted aid effectiveness. In a second study from Cape Verde, Dovlo et al. [73] noted that policy dialogues have proved to be an effective tool in health sector management and could be a crucial component of the governance dynamics of the sector. They highlighted the dialogue success factors to included: the use of innovative approaches, good facilitation, availability of resources for dialogues, good communication, and consideration of different opinions [73]. In Guinea, Kwamie and Nabyonga-Orem [74], while assessing the role of policy dialogue in evidence-informed policymaking, noted that policy dialogue improves harmonization in terms of fostering information exchange amongst partners.

#### Draft the health policy document

In drafting the policy document, it is important to keep the language simple and strait forward without losing the intent of the policy. Unnecessary jargons must be avoided with the understanding that many readers of the policy documents may not be specialists in the health issues the policy is addressing.

#### Subject the policy document to internal and external review

After drafting the policy, it is very important for the draft to be subjected to both internal and external comprehensive review process. The purpose of a comprehensive review is to take an in-depth look at the draft policy to: (i). determine if the policy is still needed or if it should be combined with another policy; (ii). determine whether the purpose and goal of the policy is achievable; (iii) determine if changes are required to improve the effectiveness or clarity of the policy; and (iv). to ensure that appropriate monitoring and evaluation mechanism is integrated into the policy [75]. The reviewers should be experts or specialists in the policy specific area and should include senior policymakers, senior researchers and senior practitioners.

#### Ensure official endorsement of the policy by government

Aligning the policymaking process with the government’s policy direction/agenda is one of the best ways to guarantee that the government in power will support and endorse the policy. There is no hope for an anti-government policy. No policy is likely to succeed to the implementation stages if it does not receive government’s endorsement. The following are four practical strategies that can be taken to facilitate government endorsement of a policy: (i). align the policy with the mandate and priority of the government in power; (ii). engage the leaders of ruling political party as champions of the policy development process; (iii). involve relevant government apparatus in all stages of the policy development; (iv). employ the process of lobbying of very influential persons in the government to drive the policy process.

Political parties the world-over are known to have strong influence on policies and policymaking process. Normally, every party has its manifesto and ideologies and these are brought into governance if the party gains power. It is important for the leaders of the political parties to be engaged in policymaking process as this strategy is likely to facilitate the policy endorsement by government of that political party. There are numerous studies that have shown that party orientation has a strong influence of policy making process and implementation [76–79].

#### Institute monitoring, evaluation and review mechanism for the policy

There is a dearth of studies focussing on policy monitoring, evaluation and reviews. Policy monitoring, evaluation and review are critical components of the evidence-informed policymaking. Every policy that is made has set objectives and desired goals or outcomes. Monitoring, evaluation and review are essential functions to ensure that the priority health actions outlined in the policy documents are implemented as planned against stated objectives and desired results [80].

To ensure that policies meet up to international best practice standard, they should have life span so as to necessitate policy review towards the expiration date. Also, regular monitoring, evaluation and review mechanism must be incorporated into the policy. According to WHO [81], regular monitoring and evaluation system is required in order to ensure that indicators are measuring what they are meant to, that data are generated according to standards, that data analysis and communication of results give the information needed by decision-makers. The main components of policy monitoring and evaluation platform include sound policy and institutional environment; well-functioning data sources; strong institutional capacity for data collection, management, analysis, use and dissemination; and effective country mechanisms for review and action [80].

## Conclusion

The production of the EBPM Guidance is a reflection of the WAHO resolve to promote evidence to policy link in the West African sub-region. Although the knowledge of evidence-informed policymaking is not yet wide spread among policymakers in West Africa, the understanding of the process is being vigorously promoted in the region. The EBPM Guidance is one of the emerging tools designed to enhance the understanding of evidence to policy process in West Africa. The strategies to facilitate the use of evidence in policymaking outlined in the Guidance, can be adapted to local context, and incorporated validated approaches that can be used to consider available evidence and its quality. These attributes are reported by Bosch-Capblanch et al. [24], as very vital for an ideal guidance for evidence-informed policies about health systems in any low-income setting.

## Data Availability

The authors confirm that all data underlying the findings are fully available without restriction upon reasonable request, which should be made to the corresponding author.
